# Clinical features of bowel disease in patients aged <50 years in primary care: a large case-control study

**DOI:** 10.3399/bjgp17X690425

**Published:** 2017-03-28

**Authors:** Sally A Stapley, Greg P Rubin, Deborah Alsina, Elizabeth A Shephard, Matthew D Rutter, William T Hamilton

**Affiliations:** University of Exeter Medical School, Exeter.; School of Medicine, Pharmacy and Health, Durham University, Wolfson Research Institute, Stockton on Tees.; Bowel Cancer UK, London.; University of Exeter Medical School, Exeter.; University Hospital of North Tees, Stockton-on-Tees.; University of Exeter Medical School, Exeter.

**Keywords:** colorectal neoplasms, diagnosis, general practice, inflammatory bowel diseases, signs and symptoms

## Abstract

**Background:**

Incidences of colorectal cancer (CRC) and inflammatory bowel disease (IBD) are increasing in those aged <50 years.

**Aim:**

To identify and quantify clinical features in primary care of CRC/IBD in those aged <50 years. This study considered the two conditions together and aimed to determine which younger patients, presenting in primary care with symptoms, would benefit from investigation for potentially serious colorectal disease.

**Design and setting:**

Matched case-control study using primary care records from the Clinical Practice Research Datalink, UK.

**Method:**

Incident cases (aged <50 years) of CRC (*n* = 1661) and IBD (*n* = 9578) diagnosed between 2000 and 2013 were each matched with up to three controls (*n* = 3979 CRC; *n* = 22 947 IBD). Odds ratios (OR) and positive predictive values (PPV) were estimated for features of CRC/IBD in the year before diagnosis.

**Results:**

Ten features were independently associated with CRC/IBD (all *P*<0.001): rectal bleeding, change in bowel habit, diarrhoea, raised inflammatory markers, thrombocytosis, abdominal pain, low mean cell volume (MCV), low haemoglobin, raised white cell count, and raised hepatic enzymes. PPVs were >3% for rectal bleeding with diarrhoea, thrombocytosis, low MCV, low haemoglobin or raised inflammatory markers; for change in bowel habit with low MCV, thrombocytosis or low haemoglobin; and for diarrhoea with thrombocytosis.

**Conclusion:**

This study quantified the risk of serious bowel disease in symptomatic patients aged <50 years in primary care. Rectal bleeding and change in bowel habit are strongly predictive of CRC/IBD when combined with abnormal haematology. The present findings help prioritise patients for colonoscopy where the diagnosis is not immediately apparent.

## INTRODUCTION

Symptoms referable to the lower gastrointestinal tract are common, accounting for 1 in 12 GP consultations.^1^ Lower gastrointestinal tract symptoms in younger patients, defined as aged <50 years, are difficult to diagnose, even after a full clinical examination. Often symptoms are attributed to non-serious conditions, such as irritable bowel syndrome (IBS), and clinical guidelines usually favour non-interventional management.^2^ However, there are more than 2300 new diagnoses of colorectal cancer (CRC) annually in the UK in patients aged <50 years.^3^ These patients are more likely than older patients to be diagnosed following an emergency presentation^4^ and have worse 5-year survival.^5^ It is unclear whether delays in diagnosis, more aggressive tumour biology, or both, are contributory factors. Furthermore, the annual incidence of inflammatory bowel disease (IBD) in the UK is approximately 13 000.^6^ A large proportion of these diagnoses are in patients aged <50 years, who often have several consultations before specialist referral.

Timely diagnosis of CRC requires clinicians to recognise the symptoms of serious bowel disease, and to investigate when appropriate. The latest National Institute for Health and Care Excellence (NICE) guidelines for suspected cancer contain recommendations for referral of younger patients aged <50 years, whereas most recommendations use an age threshold of 60 years.^2^ These recommendations for younger patients were largely based on consensus, assuming that symptoms of CRC in younger patients are similar to those in older patients. Few studies of the clinical features of CRC have included younger patients^7^^–^11 and none has reported features separately.

Inflammatory bowel diseases, primarily ulcerative colitis and Crohn’s disease, share several symptoms with CRC, notably, rectal bleeding, abdominal pain, diarrhoea, weight loss, and anaemia. Furthermore, IBD and CRC in younger patients have similar ages of onset. A systematic review in North America reported the mean age at diagnosis of Crohn’s disease to range from 33.4 years to 45 years.^12^ Mean and median ages at diagnosis of ulcerative colitis are generally 5–10 years later than those for Crohn’s disease.^13^ Annual incidence rates in the UK are 13.9 out of 100 000 for ulcerative colitis and 8.3 out of 100 000 for Crohn’s disease; thus the incidence of IBD is five to six times that of CRC.^6^ Colonoscopy and biopsy is the gold standard for diagnosis of both diseases, although calprotectin and testing for occult blood in faeces may be employed as triage tests.

For younger patients, delayed diagnosis is common for both CRC and IBD. A recent survey of 401 UK patients aged <50 years diagnosed with CRC reported that 20% presented to primary care five or more times before referral.^14^ The survey also found a strong sex divide, with 54% of males referred to a specialist after fewer than three primary care consultations compared with only 35% of females.

**How this fits in**

It is difficult for GPs to determine whether bowel symptoms in patients aged <50 years require further investigation. Delay in diagnosis of colorectal cancer (CRC) and inflammatory bowel disease (IBD) in patients aged <50 years is common and causes harm. Rectal bleeding and a change in bowel habit are both strongly predictive of CRC or IBD when combined with abnormal haematology. The CRC/IBD risk assessment tool may help to reduce diagnostic delay.

A cohort study of 1591 Swiss patients diagnosed with IBD reported the median diagnostic interval (time from first presentation of symptoms to definitive diagnosis) for Crohn’s disease to be 9 months and for ulcerative colitis, 4 months.^15^

Timely investigation is important in both conditions. In CRC, there is a clear relationship between diagnostic delay and more advanced disease, complications, and emergency presentation, and with mortality.^16^ Delayed diagnosis of IBD reduces treatment options, increases the risk of disease progression,^17^ and is psychologically detrimental.

One solution to improving the diagnosis of serious organic bowel disease in younger patients is to consider the possibility of CRC and IBD together, and to ask the clinical question, ‘Which younger patients with symptoms would benefit from investigation for potentially serious colorectal disease?’

## METHOD

This was an observational study using data collected prospectively from the Clinical Practice Research Datalink (CPRD).

The CPRD maintains records from nearly 700 participating practices in the UK.

### Identification of cases and controls

Incident cases in the CPRD between January 2000 and December 2013 were identified using a list of diagnostic medical codes (medcodes) (39 for CRC and 35 for IBD, available from the authors on request).

Patients were aged between 18 and 49 years at diagnosis and had at least 1 year of data prior to diagnosis. For the small number of patients with both conditions, the earliest condition was taken to be the index diagnosis. For each case (CRC/IBD) the CPRD randomly selected three controls matched by birth year, sex, and practice. The initial medcode for CRC/IBD was deemed to be the diagnosis date, hereafter called the index date. Controls were assigned the index date of their matched case.

Cases and controls with no consultations in the year before the index date were excluded. Controls were excluded if they had a previous diagnostic code of CRC/IBD.

### Selection of possible features of CRC/IBD

All symptoms, signs, and investigations (hereafter called ‘features’) that had previously been reported as associated with CRC/IBD were studied. These features were identified from literature reviews, clinical knowledge, patient feedback, charities, and websites. The CPRD database contains >100 000 medcodes, including several synonyms for the same symptom. These were collated into symptom ‘libraries’ and occurrences in the year before diagnosis of the case identified both for cases and controls.

Features were retained if they were reported in at least 5% of cases or controls (invariably this was in cases). Searches were conducted for abnormal laboratory investigations in the year before the index date using the local laboratory’s normal range supplied with the data.

Patients without a reported investigation were considered to be equivalent to those with a normal result. A variable ‘raised inflammatory markers’ equated to any of an abnormal erythrocyte sedimentation rate, C-reactive protein, or plasma viscosity. ‘Abnormal liver function’ encompassed raised levels of any of the hepatic enzymes included in liver function testing.

Records of varicose veins were identified to test for recording bias, assuming approximately equal prevalence in cases and controls. Calprotectin and faecal occult blood results were also sought.

### Analysis

The primary analysis used conditional logistic regression to assess the strength of association between clinical features and the combined outcome of *either* CRC or IBD. Features with *P*<0.1 in univariable analysis entered multivariable analysis.

The final model was derived from all features that survived the earlier staged regressions and used a *P*<0.01.

All analyses were performed using Stata (version 13.1).

### Power calculation and calculation of positive predictive values (PPVs)

The CPRD estimated that 12 000 cases and 36 000 controls would be provided for analysis; therefore, power, rather than sample size, calculations were performed. PPVs were derived using Bayes’ theorem, using national incidence data to estimate prior odds. These calculations are available from the authors on request.

## RESULTS

**Figure 1.**
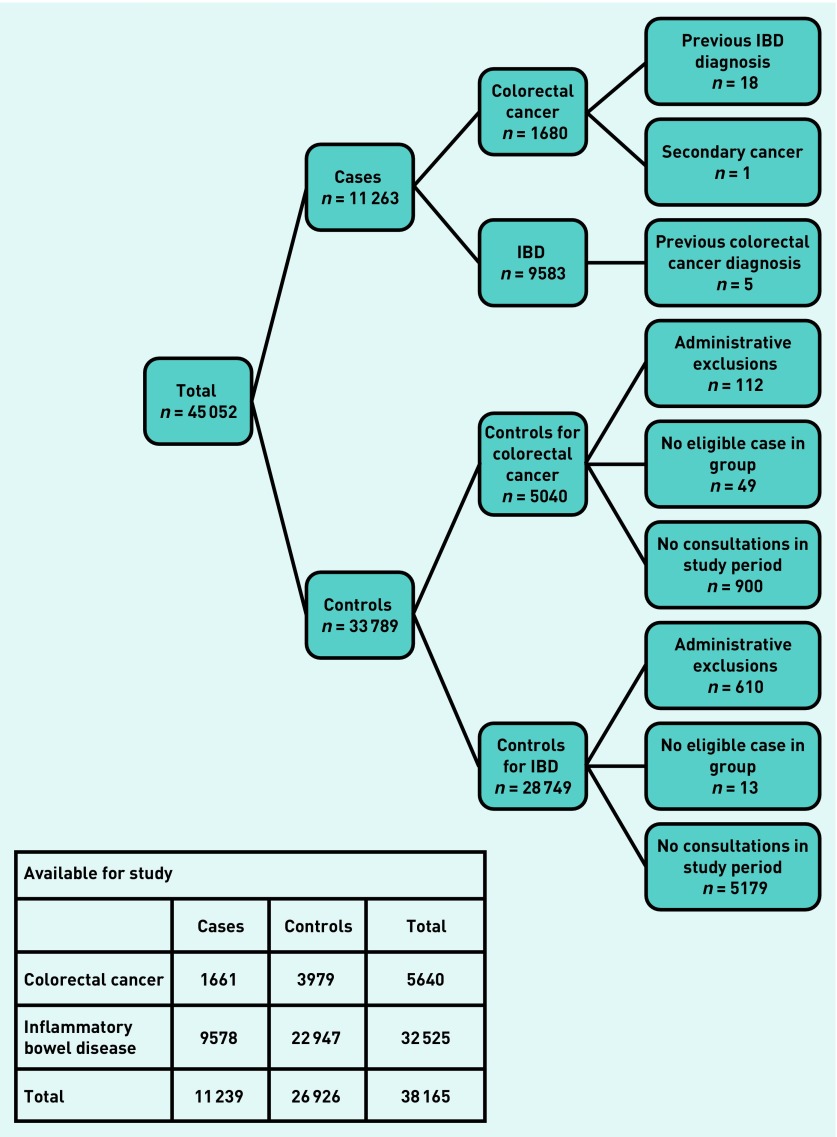
***Inclusion and exclusion of cases and controls in the combined colorectal cancer (CRC) and inflammatory bowel disease (IBD) dataset.***

The CPRD initially supplied 45 052 patients: 11 263 cases (*n* = 1680 CRC, *n* = 9583 IBD) and 33 789 matched controls. Exclusion criteria were applied (Figure 1), leading to the exclusion of one case with colonic metastatic cancer, and 23 cases with both CRC and IBD, along with their matched controls (*n* = 62). A further 722 patients (1.6%) were excluded for administrative reasons: 721 controls inadvertently reselected from the pool of cases by the CPRD, and one patient with indecipherable data. Of the remaining 44 244 patients (11 239 cases; 33 005 controls), 6079 (18.4%) controls had no consultations in the year before the index date, and were excluded. There were no cases that did not consult in the year before diagnosis. The demographic features and the consultation patterns of patients are shown in Table 1. Cases with CRC or IBD consulted more often than their matched controls in both the year and the 6 months before the index date (Mann–Whitney test: *P*<0.001).

**Table 1.** Characteristics of patients in primary care aged 18–49 years with CRC and IBD cases and matched controls

**Age, years**	**CRC**				**IBD**			
	
	**Cases *n* = 1661**			**Controls *n*= 3979**		**Cases *n*= 9578**		**Controls *n* = 22 947**

	**Total**	**Females *n*= 806**	**Males *n*= 855**		**Total**	**Females *n*= 4976**	**Males *n*= 4602**	
18–29, *n* (%)	58 (3.5)	32 (4.0)	26 (3.0)	145 (3.6)	2934 (30.6)	1518 (30.5)	1416 (30.8)	6964 (30.4)
30–39, *n* (%)	224 (13.5)	112 (13.9)	112 (13.1)	528 (13.3)	3033 (31.7)	1634 (32.8)	1399 (30.4)	7251 (31.6)
40–49, *n* (%)	1379 (83)	662 (82.1)	717 (83.9)	3306 (83.1)	3611 (37.7)	1824 (36.7)	1787 (38.8)	8732 (38)

**Mean number (SD) of consultations to primary care**								

	**CRC**				**IBD**			
	
		**Cases *n*= 1661**		**Controls *n*= 3979**		**Cases *n*= 9578**		**Controls *n* = 22 947**

	Males *n* = 855	Females *n* = 806	Males *n* = 1828	Females *n* = 2151	Males *n* = 4602	Females *n* = 4976	Males *n* = 9616	Females *n* = 13 331
1 year before diagnosis	12.1 (8.5)	15 (9.7)	5.8 (6.2)	8.3 (8.1)	12.3 (10.2)	16.4 (11)	5.2 (6.1)	8.2 (7.8)
6 months before diagnosis	9 (5.9)	10.5 (6.5)	3.3 (3.4)	4.5 (4.5)	8.4 (6.3)	10.2 (6.8)	3.1 (3.4)	4.3 (4.2)

CRC = colorectal cancer. IBD = inflammatory bowel disease.

### Associations between CRC/IBD and clinical features

Table 2 shows the frequencies of clinical features in the combined dataset, plus their likelihood ratios and positive predictive values (PPVs). All PPVs of individual features for CRC/IBD were <1%, apart from those for change in bowel habit (1%, 95% CI = 0.8% to 1.3%) and rectal bleeding (1.2%, 95% CI = 1.1% to 1.4%). In multivariable analysis, rectal bleeding (OR 42, 95% CI = 33 to 55) and change in bowel habit (OR 27, 95% CI = 19 to 38) were most strongly associated with CRC/IBD. Although constipation, nausea/vomiting, and rectal mass were independently associated with CRC (OR reported in Table 2), they occurred too infrequently (below the 5% threshold) for inclusion in the combined dataset; therefore, their positive likelihood and PPVs are not reported. Estimation of sex-specific PPVs is hampered by the IBD incidence reports often not giving separate figures. Likelihood ratios for abdominal pain, change in bowel habit, and anaemia were higher in males. The reporting of varicose veins was similar in cases (46, 0.41%) and controls (129, 0.48%) (χ^2^ test *P* = 0.354). Of 11 239 cases, 8479 (75.4%) had at least one of the features present in the final model.

**Table 2.** Clinical features in cases and controls in the whole study population and association with CRC and/or IBD

**Clinical feature**	**Frequency of features in cases and controls in CRC/IBD dataset**				**OR (95% CI) in multivariable analysis for:**		
	
	**Cases, *n* (%) *n*= 11 239**	**Controls, *n* (%) *n*= 26 926**	**Positive LR PPV^a^ (95% CI)**	**(95% CI)**	**CRC/IBD**	**IBD**	**CRC**
**Symptoms**							
Diarrhoea	3047 (27.1)	531 (2)	13.8 (12.6 to 15)	0.5 (0.5 to 0.6)	8.9 (7.5 to 11)	9.5 (7.9 to 12)	7.7 (4.3 to 14)
Abdominal pain	3040 (27.1)	1534 (5.7)	4.8 (4.5 to 5)	0.2 (0.2 to 0.2)	3.9 (3.4 to 4.5)	3.5 (3.0 to 4.1)	6.0 (4.2 to 8.7)
Rectal bleeding	2654 (23.6)	201 (0.8)	31.6 (27.5 to 36.5)	1.2 (1.1 to 1.4)	42 (33 to 55)	40 (31 to 53)	54 (26 to 110)
Change in bowel habit	730 (6.5)	65 (0.2)	26.9 (20.9 to 34.6)	1.0 (0.8 to 1.3)	27 (19 to 38)	22 (15 to 33)	58 (21 to 160)
Constipation	471 (4.2)	245 (0.9)	^a^	^a^	^a^	^a^	7.9 (4.3 to 14)
Nausea/vomiting	540 (4.8)	410 (1.5)	^a^	^a^	^a^	^a^	2.7 (1.4 to 5.1)
Rectal mass	189 (1.7)	9 (0.03)	^a^	^a^	^a^	^a^	190 (51 to 720)

**Investigations**							
Raised inflammatory markers	3115 (27.7)	575 (2.1)	13 (11.9 to 14.2)	0.5 (0.5 to 0.6)	5.4 (4.6 to 6.3)	6.1 (5.1 to 7.3)	3.1 (2.0 to 4.7)
Low haemoglobin	1802 (16)	572 (2.1)	7.6 (6.9 to 8.3)	0.3 (0.3 to 0.3)	2.5 (2.1 to 3.1)	2.2 (1.8 to 2.8)	5.2 (3.2 to 8.5)
Raised platelets	1678 (14.9)	206 (0.8)	19.5 (16.9 to 22.5)	0.8 (0.7 to 0.9)	4.4 (3.4 to 5.7)	5.4 (4.0 to 7.2)	^a^
Raised white cell count	1472 (13.1)	488 (1.8)	7.2 (6.5 to 8)	0.3 (0.3 to 0.3)	1.5 (1.3 to 1.9)	1.7 (1.4 to 2.1)	^a^
Abnormal liver function	1392 (12.4)	1019 (3.8)	3.3 (3 to 3.5)	0.1 (0.1 to 0.1)	1.4 (1.2 to 1.6)	1.4 (1.2 to 1.7)	^a^
Low mean red cell volume	1102 (9.8)	290 (1.1)	9.1 (8 to 10.3)	0.4 (0.3 to 0.4)	2.7 (2.1 to 3.5)	2.4 (1.8 to 3.2)	4.3 (2.3 to 8.0)

a *Features that did not retain independent significance in multivariable analysis in the CRC/IBD population. All associations in the above table had a* P-*value of <0.001. CRC = colorectal cancer. IBD = inflammatory bowel disease. LR = likelihood ratio. PPV = positive predictive value.*

No calprotectin results were identified. For faecal occult blood testing, there were 3026 possible entries; however, 2641 were blank, 111 positive/abnormal, and 274 normal/negative.

**Figure 2.**
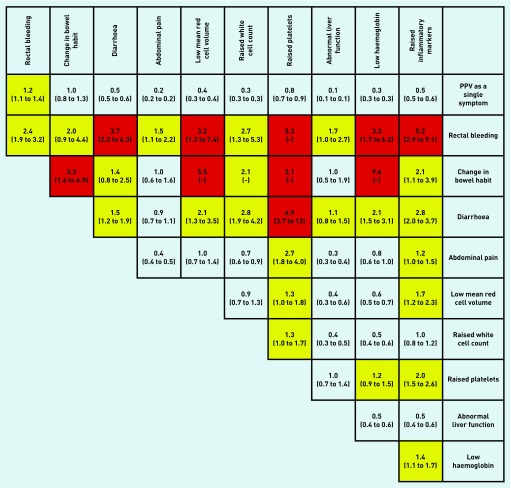
***PPVs (95% CI) for colorectal cancer (CRC) or inflammatory bowel disease (IBD) in males and females aged 18–49 years for individual risk markers and for pairs of risk markers in combination. The top figure in each cell is the PPV (95% CI) when both features are present. CI have not been calculated when any cell in the 2 × 2 table was* <*5 (invariably, this was because too few controls had both features). The yellow shading is for features with a PPV* >*1%; and the red for PPV* >*3% (see Discussion for reasoning). The cells along the diagonal relate to the PPV when the same feature has been reported twice. Thus, the abdominal pain/abdominal pain intersect is the PPV for CRC/IBD when a patient has attended at least twice with abdominal pain. PPV = positive predictive value.***

**Figure 3.**
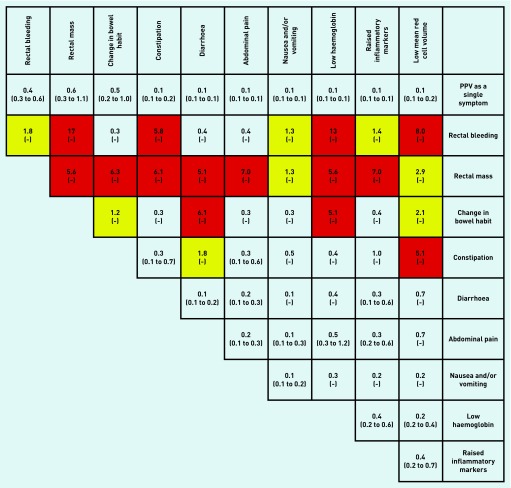
***PPVs (95% CI) for colorectal cancer (CRC) in males and females aged 18–49 years for individual risk markers and for pairs of risk markers in combination. PPV = positive predictive value.***

**Figure 4.**
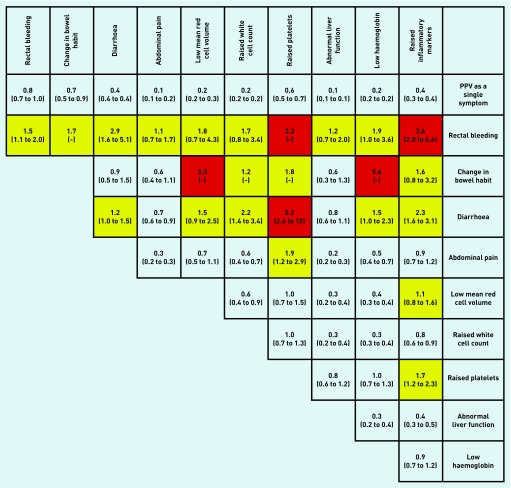
***PPVs (95% CI) for inflammatory bowel disease (IBD) in males and females aged 18–49 years for individual risk markers and for pairs of risk markers in combination. PPV = positive predictive value.***

All associations in Table 2 had a *P*-value of <0.001. Figures 2, 3, and 4 show PPVs for CRC/IBD for selected features in Table 2, and for CRC and IBD alone.

## DISCUSSION

### Summary

To the authors’ knowledge, this is the first study to examine the pre-diagnostic clinical features of CRC/IBD specifically in young-age patients. The diseases share several clinical features and a common definitive test; therefore, the primary analysis kept them together, although findings for the two diseases are also reported separately. For individual symptoms or abnormal laboratory findings, the risks of either IBD or CRC were below 1%, apart from change in bowel habit and unexplained rectal bleeding.

Pairs of symptoms, or individual symptoms accompanied by abnormal tests, however, often had risks above 1%.

### Strengths and limitations

A main strength of the present study is its data source, particularly its setting in primary care, in which the diagnostic challenge exists. CPRD data are regarded as high quality in terms of accuracy, completeness, and validity of diagnoses, and are representative of the UK as a whole. The identification of symptoms and diagnoses depended, however, on accurate primary care recording. Although there was no access to secondary care data, in particular histology, for external validation of diagnoses, cancer recording in the CPRD is deemed to be excellent.^18^ Furthermore, case ascertainment and year of diagnosis for IBD have both been validated in the CPRD.^19^

No studies have reported from primary care: unfortunately, in this study no calprotectin results and insufficient faecal occult blood results were found to allow reliable analysis. Some symptom occurrences will have been missed, because the GP either omitted to record them, or recorded them in the uncoded section of the notes. The present measures of association would only be altered materially if recording practice differed between cases and controls. Variation in recording also pertains to the three main symptoms of bowel transit: diarrhoea, constipation, and ‘change in bowel habit’. There is considerable overlap between the first two of these and the last. It was chosen not to merge these into one variable, as it would have obscured differences between constipation and diarrhoea.

The present methodology is well accepted, in particular the derivation of likelihood ratios within the study, accompanied by the use of external incidence data to estimate the PPVs.^20^ The prior odds part of the PPV calculation requires accurate incidence figures: this was simple for colorectal cancer, with high-quality national figures being published annually, but less so for IBD. The present choice of a median incidence figure from recent studies from Northern Europe sought to match the present dataset, but should the true UK incidence of IBD in this age group be higher (or lower) than the 22 out of 100 000 (IQR 15.25–28) used, the PPVs would be commensurately higher or lower too.

Finally, the study design matched the clinical problem: namely, which symptomatic patients in primary care warrant investigation? The coupling of the two diseases analytically is therefore a strength rather than a limitation.

### Comparison with existing literature

To the authors’ knowledge, no previous study has considered the two diseases together for the purpose of clinical decision making; furthermore, very little literature describes the primary care symptomology of either disease in this age group. Studies from secondary care are generally unhelpful because they report on a referred population: in general, such studies report much higher PPVs for symptoms of cancer (and presumably would do for IBD) than do those from the primary care population.^21^

### Implications for research and practice

Younger patients with lower gastrointestinal tract symptoms represent a difficult diagnostic problem for GPs, even after a full clinical examination. Most will have functional or non-serious organic disease, and most of those who undergo colonoscopy have no relevant abnormality.^22^ Consequently, less invasive symptom assessment strategies to differentiate functional from organic disease are preferred.^23^^,^^24^ Faecal calprotectin has a diagnostic role in differentiating between inflammatory and non-inflammatory bowel conditions such as IBS. In a recent systematic review, using a cut-off of 50 µg/g, the pooled sensitivity and specificity of faecal calprotectin for IBD were 93% and 94%, respectively.^25^ Faecal calprotectin is not recommended, however, for discriminating between functional gut disease (such as IBS) and CRC. A recent report using 654 patients referred for possible CRC reported that calprotectin had a negative predictive value of 98.6% for cancer, or 97.2% when polyps ≥10 mm were included.^26^ Future studies incorporating a calprotectin test result would be valuable to examine what predictive power they add to the present PPVs.

The present results support a diagnostic strategy in primary care outlined in NICE CG61.^23^ It is predicated on an initial physical examination, including rectal examination, and baseline full blood count and inflammatory marker level in patients aged <50 years presenting with symptoms referable to the large bowel. For those whose diagnosis is not immediately apparent, the CRC/IBD risk assessment tool (Figure 2) guides subsequent action. It is considered that those with a risk of CRC/IBD >3% warrant referral for urgent colonoscopy or specialist assessment, an action compatible with both NICE NG12 and the NICE Quality Standard for IBD (QS81).^2^^,^^27^ Patients with a risk of 1–3% should have their calprotectin level estimated, as IBD is the more likely of the two diagnoses and can be excluded by a normal calprotectin level. Those with a risk below 1% can be managed expectantly. In the latter two scenarios, it is important that the patient’s progress is monitored (safety-netting) and that referral is made if clinical progress is not as expected. Use of the risk assessment tool also allows a more nuanced decision on referral of patients in this age group who present with unexplained rectal bleeding, going beyond the blanket recommendation of referral in NICE CG61.^23^ The tool has been colour-coded to mirror these suggestions.

The present risk assessment tool is limited to risk of CRC/IBD. For females whose symptoms could represent ovarian cancer, the risk assessment tool for that cancer site should also be employed.^28^ For those patients whose symptoms could represent coeliac disease, the British Society of Gastroenterology guidance on diagnostic assessment should be followed.^29^

This strategy should increase the proportion of younger patients with inflammatory bowel disease or colorectal cancer to be diagnosed without delay. It seeks to do so without inordinately increasing work for colonoscopy providers, who are already experiencing considerable increases in workload.^30^ What is missing is a health-economic perspective. It is possible to estimate the costs of increased colonoscopies but much more difficult to estimate cost-effectiveness. Until this economic analysis is complete, investigation strategies are limited to being based on likely clinical benefits; which include avoiding colonoscopy in those with the least to benefit as well as offering it for those with the most to benefit.
